# Supplementary material for: A Global Screen for Assembly State Changes of the Mitotic Proteome by SEC-SWATH-MS
Source: Cell Syst. 2020 Feb 26;10(2):133–155.e6. doi: 10.1016/j.cels.2020.01.001 (PMC7042714; doi:10.1016/j.cels.2020.01.001)

Q8IZA0 | K319L_HUMAN | KIAA0319L AAVR KIAA1837 PP791
Monomer MW [kDa]: 115.658 Monomer expected elution fraction: 39

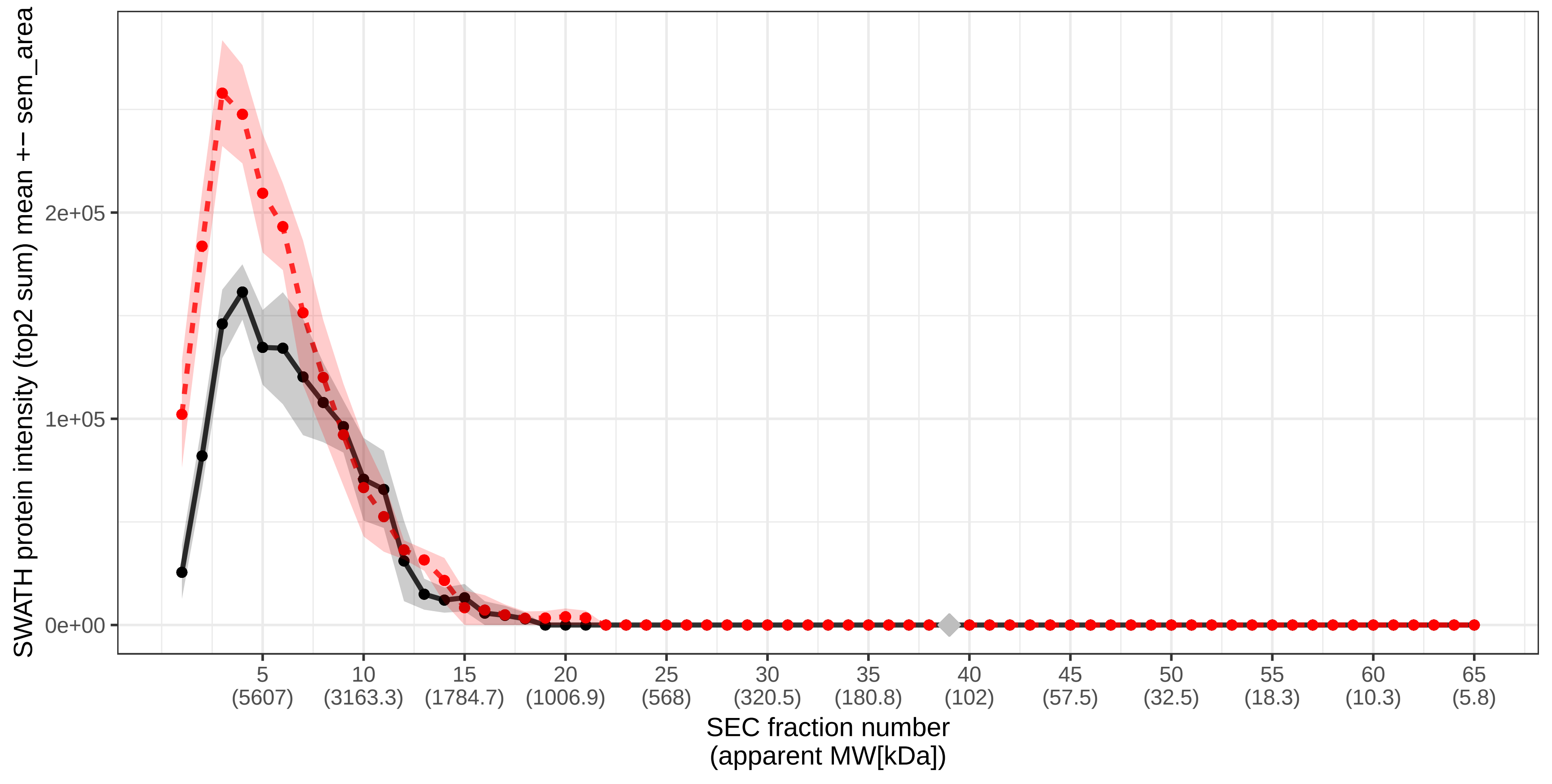

Supplement: Data S1. SEC-SWATH-MS Protein Chromatograms, Related to Figure 1 [file mmc6.zip › SECchrom_Q8IZA0_K319L_HUMAN_KIAA0319L_AAVR_KIAA1837_PP791.pdf]
